# Body experience influences lexical-semantic knowledge of body parts in children with hemiplegic cerebral palsy

**DOI:** 10.3389/fpsyg.2022.955939

**Published:** 2022-09-07

**Authors:** Thalita Karla Flores Cruz, Deisiane Oliveira Souto, Korbinian Moeller, Patrícia Lemos Bueno Fontes, Vitor Geraldi Haase

**Affiliations:** ^1^Graduate Program in Neuroscience, Universidade Federal de Minas Gerais, Belo Horizonte, Brazil; ^2^Laboratório de Neuropsicologia do Deselvolvimento (LND), Universidade Federal de Minas Gerais, Belo Horizonte, Brazil; ^3^Centre for Mathematical Cognition, School of Science, Loughborough University, Loughborough, United Kingdom; ^4^Leibniz-Institut für Wissensmedien, Tübingen, Germany; ^5^LEAD Graduate School and Research Network, University of Tübingen, Tübingen, Germany; ^6^Individual Development and Adaptive Education Center, Frankfurt, Germany; ^7^Department of Physiotherapy, Pontifícia Universidade Católica de Minas Gerais, Belo Horizonte, Brazil; ^8^Department of Psychology, FAFICH, Universidade Federal de Minas Gerais, Belo Horizonte, Brazil

**Keywords:** body representation, body image, sensorimotor experience, neuropsychology, hemiplegic cerebral palsy

## Abstract

**Background:**

Disorders in different levels of body representation (i.e., body schema, body structural description, and body image) are present in hemiplegic cerebral palsy (HCP). However, it remains unclear whether the body image develops from aspects of body schema and body structural description, and how this occurs in children with HCP.

**Objective and methods:**

In a cross-sectional study, we investigated 53 children with HCP (mean age about 10 years) and 204 typically developing (TD) control children to qualitatively evaluate whether and how body schema (related sensorimotor experiences) and body structural description (related visuospatial experiences) affect the development of children’s body image and whether this development is delayed through HCP. Graph analysis was used to create a lexical-semantic map of body representation from data of a semantic word fluency task.

**Results:**

Results indicated a similar qualitative pattern of influences of sensorimotor and visuospatial experiences on lexical-semantic knowledge of body parts, with a delayed developmental course in children with HCP compared to TD children.

**Conclusion:**

These findings suggest that children’s body image seemed to be influenced by body schema and body structural descriptions as indicated by poorer lexical-semantic knowledge of body parts in children with HCP due to missing physical experiences of the affected body parts. This might imply that “body talk” may beneficially complement physical therapy for children with HCP to promote body image development.

## Introduction

Children with hemiplegic cerebral palsy (HCP) learn strategies to manage their everyday life using only one hand as the affected limb is usually being neglected or not used – a phenomenon known as “developmental disregard” (Houwink et al., 2013). They may present functional limitations related to the affected upper limb that cannot be explained by muscle strength impairments and may be aggravated by visuoperceptual disorders (de Ajuriaguerra and Stucki, 1969). Additionally, unilateral neglect may further impair the processing of perceptual information from the environment (Katz et al., 1998). Interestingly, children with HCP often also show atypical processing of information associated with their body, resulting in sensory deficits in their upper extremities such as threshold disturbances in proprioception as well as the perception of touch and pain (Riquelme and Montoya, 2010). To account for these symptoms, the hypothesis of a disorder of higher-level body representation in HCP was proposed by DeAjuriaguerra and Stucki decades ago (de Ajuriaguerra and Stucki, 1969). Taken together, one might speculate that some symptoms observed in children with HCP may be due to an impairment of body representation at different levels (Fontes et al., 2014).

Body representations have been suggested to be organized into three neuropsychological levels: sensorimotor, visuospatial, and semantic-lexical (Sirigu et al., 1991; Goldenberg, 2003). The sensorimotor representation of the body, henceforth, referred to as body schema, incorporates proprioceptive information about the body itself and is characterized by continuous updating and consequent adaptation to changes in body properties and relative positions due to movements (Sirigu et al., 1991; Coslett et al., 2002; Goldenberg, 2003). The visuospatial representation, also termed and henceforth referred to as body structural description, describes the topographical representation of the body, providing information about its shape and surface contours as well as continuity and proximity relations among different body parts (Sirigu et al., 1991; Coslett et al., 2002; Goldenberg, 2003). Finally, body-related semantic-lexical knowledge is part of what we henceforth refer to as body image, which includes general information about names of body parts, associations of body parts with tools and artifacts, functions of different body parts, and affective information about the body (Sirigu et al., 1991; Coslett et al., 2002; Goldenberg, 2003).

Contrary to the extensive literature on representational deficits regarding the body in adults with (unilateral) brain damage, only a few studies investigated impairments of body representation in brain-damaged children (Frassinetti et al., 2012; Fontes et al., 2014, 2016; Corti et al., 2018; Butti et al., 2019; Di Vita et al., 2020). Of those, three evaluated all levels of body representation in children with cerebral palsy (Fontes et al., 2014, 2016; Di Vita et al., 2020) whereas two of them only considered children with HCP (Fontes et al., 2014, 2016). The results of Fontes et al. (2014) suggest that similar to adult stroke patients, impairments of body representation in children with HCP are related to a decrease in spontaneous use of the affected limb not explained by motor problems directly associated with the respective brain damage (Fontes et al., 2014). Fontes et al. (2016) reported evidence substantiating those damages to the immature brain, such as HCP, which seem to drive disorders in all three levels of body representation (Fontes et al., 2016). “Interestingly, by categorizing 5- to 11-year old children into two age groups (5–7 and 8–11 years old), Di Vita et al. (2020) found alterations in different body representations in children with cerebral palsy (including the three main types of cerebral palsy: hemiplegia, diplegia, and quadriplegia) at specific developmental stages. In particular, when compared to typically developing (TD) children of the same age, children with cerebral palsy aged from 5 to 7 years old did not show significant differences in body representation tasks (Di Vita et al., 2020). However, the group of older children with cerebral palsy (aged 8–11 years) showed significant differences in body structural description and body schema, but not body image (related to body semantics) (Di Vita et al., 2020). These findings might be explained by the fact that body structural description and body schema were not yet fully developed in TD children with 5–7 years old (Raimo et al., 2021).”

Impairments of different levels of body representation are dissociable in adults with brain damage, (Sirigu et al., 1991; Schwoebel and Coslett, 2005; Raimo et al., 2022) but interact. The latter is inferred from the observation that body structural description was observed to influence body schema in experiments using the rubber hand illusion (Botvinick and Cohen, 1998). Moreover, in TD children aged between 5 and 10 years, body structural description was observed to influence body image as indicated by children’s naming performance for the location of the body parts (e.g., body parts *vs.* head features and also upper *vs.* lower limbs) or their involvement in motor skills (e.g., distal segments, joints, and broader body parts) (Auclair and Jambaqué, 2014). Furthermore, performance on tasks assessing body structural description (e.g., finger gnosia, verbal and visual body parts localization, matching body parts by location) was found associated positively with performance on tasks measuring body schema (e.g., imitation of meaningful and meaningless gestures) in a study investigating and comparing TD children and children with HCP (Fontes et al., 2016). Also, performance on body image tasks (e.g., naming body parts) was associated positively with performance on tasks measuring body structural description (e.g., finger gnosia, verbal and visual body parts localization, matching body parts by location) and body schema tasks (e.g., hand laterality judgment task and imitation of meaningful gestures) (Fontes et al., 2016). Against the background of this brief overview of the literature, it seems that body representations develop in a more or less hierarchical manner with body structural description gradually developing based on body schema, and body image gradually developing from body structural description.

However, little is known so far about whether and how body representations of children with HCP develop as compared to TD children. In particular, there are only a few investigations of how an injury to the immature brain may impact the development of body representations during childhood (Christie and Slaughter, 2009; Simons and Dedroog, 2009; Simons et al., 2011; Frassinetti et al., 2012; Fontes et al., 2016; Corti et al., 2018; Butti et al., 2019; Di Vita et al., 2020). Therefore, this study investigated the development of body image using a word fluency task comparing the performance of TD children with that of children with HCP. We were particularly interested in whether body schema (related sensorimotor information) and body structural description (related visuospatial information) contribute to the development of body image (by qualitatively analyzing the body parts most cited in the word fluency task), and whether this development is delayed in children with HCP. As such, we compared performance on word fluency not only for body parts but also for animals, based on lexical-semantic maps using Graph Analysis across different age groups and comparing TD children and children with HCP.

## Materials and methods

### Participants

This cross-sectional study involved a convenience sample of children with a diagnosis of HCP recruited in rehabilitation centers in Minas Gerais (Brazil). TD control children were recruited from public and private schools in Minas Gerais (Brazil). Children eligible to participate in the study met the following inclusion criteria: (i) performance above the 15*^th^* percentile in the assessment of general cognitive ability, (ii) no uncontrolled epilepsy, and (iii) ability to respond to the assessment procedures. The sample comprised 257 children in total, of which 204 were TD control children (age range 4–12 years, mean age = 8.09 years, SD = 2.60 years) and another 53 children with HCP [age range 7–12 years; mean age = 10.19 years, SD = 1.83 years; 36 right hemiplegic cerebral palsy (RHCP) and 17 left hemiplegic cerebral palsy (LHCP)]. To evaluate a potential delay in development of body image, we compared performance of children with HCP to that of TD children separated into three age groups: (i) 4–6 years (*n* = 69; mean age = 5.40 years, SD = 0.72 years), (ii) 7–9 years (*n* = 59; mean age = 8.89 years, SD = 1.03 years), and (iii) 10–12 years (*n* = 76; mean age = 11.21 years, SD = 0.96 years).

### Ethics

This study was approved by the Research Ethics Committee of the Federal University of Minas Gerais (protocol number 2.155.379). Participation was conditioned to get written informed consent from parents or legal guardians, and oral consent from children.

### Materials

#### General cognitive abilities

General cognitive abilities were assessed using the Raven’s Progressive Coloured Matrices (RCPM) (Angelini et al., 1999) validated for the Brazilian population. Children who scored below 15_*th*_ percentile were not considered for the study. Analyses considered z-scores (*M* = 0, SD = 1), computed as described in the test manual.

#### Semantic word fluency task

The Semantic Word Fluency task evaluates the spontaneous production of words under restricted search conditions (Strauss et al., 2006). In two runs, each child had to produce as many animals in one and body parts in the other run, respectively, within 60 s each. We recorded the total number of words produced, the total number of categorically correct words produced, the total number of repetitions, and the total number of intrusion errors as measures of children’s performance. The number of categorically correct and repeated words was considered the dependent variable in the graph analysis.

### Procedure

Data collection took place in schools and rehabilitation centers that children attended. Assessment of general cognitive abilities and application of the Semantic Word Fluency task was carried out by a team of trained undergraduate students in one-on-one sessions lasting about 40 min per child.

### Graph analysis

The sequence of words produced in the Semantic Word Fluency task was represented as an individual graph using *SpeechGraphs* software (Mota et al., 2012). The graphical structure reflects associations between a set of items expressed in the form of a network composed of nodes and edges, where nodes represent the items (i.e., words produced by children) and the edges the connections between these items (Albert and Barabasi, 2002; Mota et al., 2012). In addition to the sum of categorically correct words as well as repetitions (number of correct words and number of repetitions – CWR) obtained from the verbal fluency task, the software estimated six attributes: (i) number of nodes (N), (ii) number of edges (E), (iii) density (D – number of edges divided by the number of possible edges), (iv) diameter (DI), and (v) average shortest path (ASP – the shortest path length between pairs of more distant nodes in a network) (Albert and Barabasi, 2002). Better semantic networks would be indicated by *N* − 1 edges of low density and with great distances, thereby generating direct graphs. When words were repeated, the graphs generated presented E ≥ N and high density. In addition to individual graphs, group graphs were created to reflect semantic networks of children with HCP and the three age groups of TD children. Semantic network scores for body parts were used to identify the most frequent or typical words, which were then used for further analyses.

### Statistical analyses

Preliminary analyses indicated that children with left HCP and right HCP did not differ in their scores on general cognitive ability as well as semantic word fluency. Therefore, these two groups were pooled for the analyses. In the next step, analyses of variance (ANOVA) were conducted to evaluate differences in general cognitive abilities between the group of children with HCP and the three different age groups of TD children.

For the Semantic Word Fluency task, group differences in the number of correct words, repeated words, and errors, as well as parameters obtained from the graph analysis, were analyzed using mixed model analyses of covariance (ANCOVA) discerning the between-participant factor group (i.e., children with HCP *vs.* the three different age groups of TD children) and stimulus category (i.e., animals *vs.* body parts) while controlling for influences of general cognitive abilities. Additionally, we evaluated performance in the word fluency task using within-participant repeated measures ANOVA discerning the number of correct animals and number of correct body parts for each participant group.

We also explored the effects of body schema and body structural description on body image by qualitatively analyzing words that composed the semantic network nuclei for the four groups.

## Results

### Differences in general cognitive abilities between groups

Despite all children scoring above percentile 15, the ANOVA revealed a significant effect of the participant group on children’s scores for general cognitive ability (*F_(4;256)_* = *7.945; p* < *0.01;* η^2^*_*p*_* = *0.11*). Bonferroni corrected pair-wise comparisons indicated that children from the HCP group (*M* = −0.03, SD = 0.49) had significantly lower scores than the three TD groups (*all p* < *0.001*; TD 4–6 years: *M* = 0.55, SD = 0.89; TD 7–9 years: *M* = 0.62, SD = 0.68; and TD 10–12 years: *M* = 0.54, SD = 0.64). In contrast, there was no significant difference between any two of the three groups of TD children (*all p* > *0.05*). Therefore, we considered general cognitive ability as a control variable in our subsequent analyses.

### Semantic word fluency task

#### Number of categorically correct words produced

The mixed model ANCOVA revealed a significant main effect of group for the number of correct answers. Table 1 provides statistical details and descriptive results. *Post hoc* pairwise comparisons indicated that there was no significant difference between children with HCP and TD 4-6 years for both animals and body parts (*p* > *0.45*). Children from the TD 7–9 and TD 10–12 groups produced more animals and body parts than children with HCP and those from the TD 4–6 group (all *p* < *0.001*). Finally, children from the TD 10–12 group produced more animals and body parts than children from the TD 7–9 group (*p* < *0.001*).

**TABLE 1:** Results of the semantic word fluency task.

	TD 4–6 years	TD 7–9 years	TD 10–12 years	HCP	F (3; 252)	*P*	Partial η 2	*Post hoc* (Bonferroni test)
		
	Mean (sd)							
**Animals**								
Correct words	9.22 (3.31)	12.81 (3.36)	15.38 (3.74)	9.94 (2.82)	46.726	< 0.01	0.357	HCP = TD 4–6 years < TD 7–9 years < TD 10–12 years.
Repetitions	0.94 (1.40)	0.75 (1.35)	0.58 (1.36)	0.49 (0.75)	2.164	< 0.09	0.025	–
Errors	0.16 (0.47)	0.05 (0.22)	0.01 (0.11)	0.04 (0.19)	3.359	< 0.01	0.038	HCP = TD 4–6 years = TD 7–9 years; HCP = TD 7–9 years = TD 10–12 years; TD 4–6 years > TD 10–12 years.
**Body parts**								
Correct words	10.26 (3.31)	14.15 (3.45)	16.61 (4.40)	10.47 (3.52)	43.288	< 0.01	0.340	HCP = TD 4–6 years < TD 7–9 years < TD 10–12 years.
Repetitions	0.75 (1.02)	1.14 (1.49)	0.66 (1.09)	0.60 (0.86)	2.784	< 0.08	0.032	–
Errors	0.49 (1.14)	0.17 (0.37)	0.07 (0.25)	0.06 (0.23)	7.198	< 0.01	0.079	TD 4–6 years > TD 7–9 years = TD 10–12 years = HCP.

Comparisons between typically developing children group (TD groups: TD 4–6 years, TD 7–8 years, TD 10–12 years) and children with hemiplegic cerebral palsy (HCP). TD 4–6 years, Typically developing children group (4–6 years old); TD 7–9 years, Typically developing children group (7–9 years old); TD 10–12 years, Typically developing children group (10–12 years old); HCP, hemiplegic cerebral palsy; sd, standard deviation; F, ANCOVA’s ratio F; partial η2, partial eta squared.

Additionally, the main effect of the stimulus category was significant indicating that overall children produced more body parts than animals within the respective 60 s runs (Table 2). Interestingly, simple effects for the individual groups indicated that this was only the case for all TD control groups (all *p* < *0.02*), but not for children with HCP (*p* = *0.13*). Additional Bayesian analysis following the recommendations by Masson (2011) of the posterior probability substantiated that there was no difference between the number of animals and body parts produced by children with HCP (> *0.63* probability) by providing weak evidence in favor of the null hypothesis. The interaction of group and stimulus category was not significant though.

**TABLE 2:** Comparison between animals and body parts production (correct words).

Groups	Animals	Body parts	F	*P*	Partial η ^2^
		
	Mean (sd)				
HCP	9.94 (2.82)	10.47 (3.52)	2.308	< 0.135	0.043
TD 4–6	9.22 (3.31)	10.26 (3.31)	5.935	< 0.017	0.080
TD 7–9	12.81 (3.36)	14.15 (3.45)	9.251	< 0.004	0.138
TD 10–12	15.38 (3.74)	16.61 (4.40)	6.317	< 0.014	0.078

TD 4–6 years, Typically developing children group (4–6 years old); TD 7–9 years, Typically developing children group (7–9 years old); TD 10–12 years, Typically developing children group (10–12 years old); HCP, hemiplegic cerebral palsy; sd, standard deviation; F, ANCOVA’s ratio F; partial η2, partial eta squared.

Finally, the covariate significantly influenced the results for the number of correct answers for both animals (*p* < *0.02*) and body parts (*p* < *0.01*) with children with higher general cognitive ability producing more correct answers.

#### Repetitions

There was no significant difference neither between groups nor for stimulus category for the number of repetitions with the respective main effects being not significant. Additionally, the interaction was also not significant. The covariate was not significant for the number of repetitions for both animals (*p* > *0.06*) and body parts (*p* > *0.51*).

#### Number of errors committed

There was a significant main effect of group for errors committed. *Post hoc* pairwise comparisons indicated that there was no significant difference between the number of errors committed by children with HCP and children in the TD 7–9 years and 10–12 years age groups for both animals as well as body parts (all *p* > *1.00*). The number of errors committed by children with HCP and children in the 4–6 years for animals’ categories was not significant (*p* > *0.29*). However, the number of errors committed by children with HCP was significantly lower than the number of errors committed by TD children in the 4–6 years group for body parts categories (*p* < *0.001*). The number of errors committed by TD children in the 4-6 years group was significantly higher than the number of errors committed by TD children in the 10-12 years group for the animals’ category (*p* < *0.04*), and higher than the number of errors committed by TD children in the 7-9 TD 7-9 years and 10-12 years age groups (all *p* < *0.03*). There was no significant difference in the number of errors committed by TD 7–9 years and 10–12 years age groups (*p* > *1.00*). The interaction was not significant. The covariate was also not significant for the number of repetitions for both animals (*p* > *0.4*) and body parts (*p* > *0.20*).

### Graph parameters

The mixed model ANCOVA revealed a significant main effect of group for all parameters (see Table 3). Bonferroni-corrected pairwise comparisons indicated the three TD groups differed significantly from each other with respect to the number of nodes, density, diameter and mean of the shortest path (all *p* < *0.03*). For children of the TD 10–12 years group graphs were significantly less dense with a higher number of nodes and edges, larger diameters, and ASP than for children of the TD 7–9 years and TD 4–6 years groups. The group TD 7–9 years presented intermediate parameters, which differed significantly from all parameters presented in the other TD age groups. The graphical parameters obtained for the HCP group differed significantly from the parameters obtained for the TD 7–9 years and TD 10–12 years groups (all *p* < *0.001*), but showed no significant difference from parameters observed for the TD 4–6 years group. The interaction was not significant. The covariate was also not significant for the graph parameters (all *p* > *0.7*).

**TABLE 3:** Comparisons among groups in word fluency task (graph analysis of body parts category).

	TD 4– 6 years	TD 7– 9 years	TD 10– 12 years	HCP group	F (3;252)	*P*	Partial η ^2^	*Post hoc* (Bonferroni test)
					
	Mean (sd)							
Nodes	10.23 (3.07)	14.12 (3.60)	16.58 (4.14)	10.13 (3.34)	48.792	< 0.001	0.367	HCP = TD 4–6 years < TD 7–9 years < TD 10–12 years
Edges	9.88 (3.44)	13.90 (3.85)	16.20 (4.45)	9.72 (3.62)	41.663	< 0.001	0.332	HCP = TD 4–6 years < TD 7–9 years < TD 10–12 years
Density	0.21 (0.07)	0.16 (0.05)	0.13 (0.03)	0.23 (0.08)	33.493	< 0.001	0.285	HCP = TD 4–6 years > TD 7–9 years > TD 10–12 years
Diameter	7.80 (2.83)	10.93 (4.07)	13.29 (4.57)	7.53 (2.81)	34.643	< 0.001	0.292	HCP = TD 4–6 years < TD 7–9 years < TD 10–12 years
Average Shortest Path	3.29 (0.97)	4.39 (1.33)	5.19 (1.48)	3.22 (0.94)	37.861	< 0.001	0.311	HCP = TD 4–6 years < TD 7–9 years < TD 10–12 years

TD 4–6 years, Typically developing children group (4–6 years old); TD 7–9 years, Typically developing children group (7–9 years old); TD 10–12 years, Typically developing children group (10–12 years old); HCP group, hemiplegic cerebral palsy group; sd, standard deviation; F, ANCOVA’s ratio F; partial η2, partial eta squared.

To substantiate the observed null effect for the differences between the children with HCP and those from the TD 4–6 year group, we again conducted Bayesian analysis as recommended by Masson (2011). The comparison of the HCP group with the TD 4–6 years group revealed > *0.89* probability and thus positive evidence in favor of the null hypothesis (Table 4) according to classification guidelines proposed by Masson (2011).

**TABLE 4:** Bayesian analysis investigating non-significant differences between the HCP and TD 4–6 year groups.

Graph parameter	HCP	TD 4−6 years	*df*	*SS*_*effect*_	*SS*_*error*_	*F*	*BF*	*p*_*BIC*_(H_0_| D)
Nodes	10.13 (3.34)	10.23 (3.07)	1; 119	4.516	1175.270	0.457	8.68302409	0.89672648
Edges	9.72 (3.62)	9.88 (3.44)	1; 119	3.816	1433.889	0.317	9.3131287	0.90303621
Density	0.23 (0.08)	0.21 (0.07)	1; 119	0.001	0.741	0.131	10.0675661	0.9096459
Diameter	7.53 (2.81)	7.80 (2.83)	1; 119	0.897	940.861	0.113	10.3074047	0.91156238
Average Shortest Path	3.22 (0.94)	3.29 (0.97)	1; 119	0.131	106.801	0.146	10.1413887	0.91024458

SSeffect, sum of squares for the effect; SSerror, sum of squares for errors; F, ANCOVA’s ratio F; BF, Bayes factor; p_BIC_(H_0_| D), posterior probability generated by bayesian information criterion (BIC).

### Semantic network cores

**FIGURE 1:**
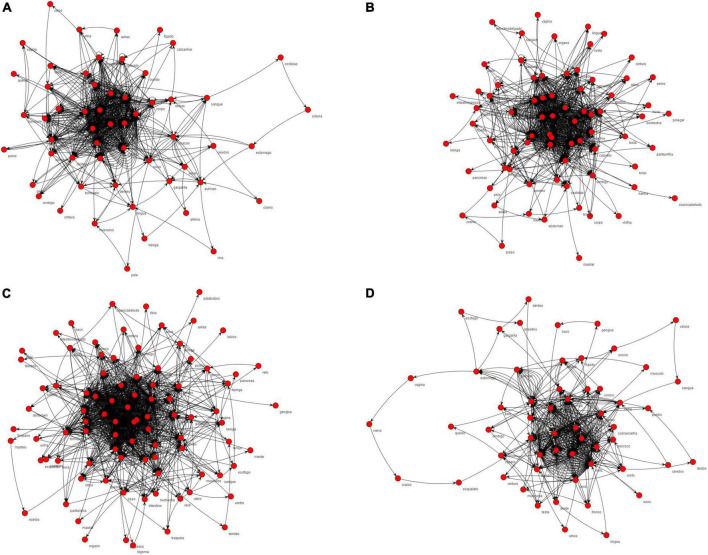
Semantic networks of body parts category formed by the groups. In **(A)** semantic network formed by TD 4–6 years; **(B)** semantic network formed by TD 7–9 years; **(C)** semantic network formed by TD 10–12 years; **(D)** semantic network formed by HCP group.

**FIGURE 2:**
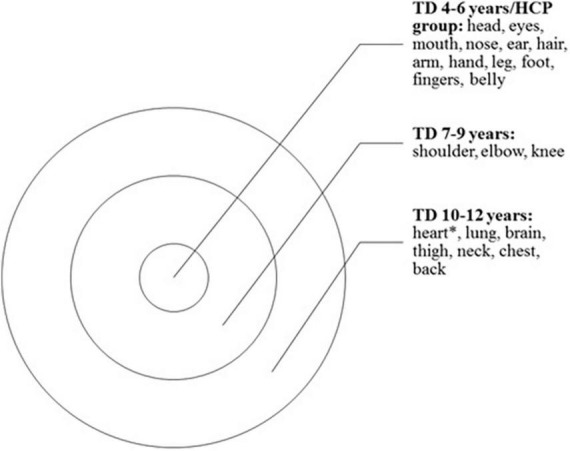
Illustration representing the semantic network core obtained from networks, representing the words quoted more frequently for each category. All groups presented a common central core. TD 4–6 years, Typically developing children group (4–6 years old); TD 7–9 years, Typically developing children group (7–9 years old); TD 10–12 years, Typically developing children group (10–12 years old); HCP group, hemiplegic cerebral palsy group; *heart was also cited by HCP group.

The networks formed by the HCP group and TD 4–6 years, TD 7–9 years, and TD 10–12 years groups and the semantic nuclei obtained from the networks, which represent the words quoted more frequently for each category, are shown in Figures 1, 2. All groups presented a common central semantic network core.

## Discussion

The current study aimed to investigate the development of body image using a word fluency task and compare the performance of TD children on this task to the performance of children with HCP. We not only evaluated performance on word fluency for body parts but also for animals, based on lexical-semantic maps of the body image generated with Graph Analysis across different age groups and between TD and children with HCP. Apart from quantitative differences between groups and stimulus categories, we were also interested in examining (by qualitatively analyzing the body parts most cited) whether body schema (related sensorimotor information) and body structural description (related visuospatial information) contribute to the development of body image, and whether this development is delayed in HCP.

Children with HCP presented a representational profile of body image (as reflected by their performance in the semantic fluency task), which seemed equivalent to that of children from the TD 4–6 years group. However, they performed significantly worse than TD children of the other age groups including those of the same age. This may reflect a continuing maturation of body image in TD children not seen in children with HCP in a comparable manner. These results will be discussed in more detail in the following.

### Semantic word fluency in hemiplegic cerebral palsy children

Children with HCP (aged from 7 to 12 years old) performed significantly worse than TD children 7–9 years and 10–12 years of age as regards the number of correct words produced, related to the retrieval of the semantic memory content. This is consistent with previous findings (Carlsson et al., 1994; Kolk and Talvik, 2000). Interestingly, they performed comparably to the youngest TD group (i.e., 4–6 years of age) as substantiated by Bayesian analyses. In relation to the number of errors and repetitions in semantic word fluency [reflecting influences of executive functions (Anderson, 2002), children with HCP did not perform significantly differently than TD 7–9 years and TD 10–12 years. This contrasts with previous research, which observed impairment of executive functions (evaluated by verbal fluency) following early brain injury (Bodimeade et al., 2013). In addition, there was no evidence for differences in semantic verbal fluency according to the side of hemiplegia (Bodimeade et al., 2013). According to a recent review, results regarding the presence of language impairments in children with HCP are inconclusive and whether they are observed might be due to differences in the neural reorganization, and in location and extent of neural lesions (Liegeois et al., 2004; Bottcher, 2010).

Regarding the potential differential influence of HCP on verbal fluency for animals and body parts, our results indicated a significant difference in the number of body parts and animals produced, with more body parts than animals produced overall. However, this difference was only significant for the control groups (TD 4–6, TD 7–9, and TD 10–12 years). For the HCP group, this advantage for body parts was not observed. This may reflect a specific relative impairment for the representation of body parts due to HCP.

### Structural characteristics of lexical-semantic body representation networks

Graph-theoretical analyses revealed qualitatively similar profiles for children with HCP and TD children. The qualitative conservation of the basic graphical properties across the four groups seems to suggest that basic mechanisms of categorical fluency might be similar (Strauss et al., 2006; Vitevitch, 2008). This also implies that connections formed may not be random, because some nodes presented many connections and many more nodes had few only connections, characterizing a free scale network. Free scale networks emerge from growth and preferential attachment mechanisms. Growth refers to the addition of new nodes (reflecting words cited) to the network over time (Vitevitch, 2008). Preferential attachment is a constraint that makes it more likely for new nodes being added to the system to connect to nodes that are already highly connected (Vitevitch, 2008). In terms of words, it means that a new word included in the networks will be probably connected to the words that were produced more often previously.

Overall, the performance of children with HCP was quite similar to that of the group TD 4–6 years with quantitative parameters suggesting a lower degree of complexity of their networks than those presented by TD children older than seven years. Also, the semantic networks produced by TD 7–9 years and TD 10–12 years groups were more direct (with less repetition of words), resulting in less dense networks. In addition to the larger vocabulary of older children their networks probably also reflect the establishment of functional relations between body parts (e.g., feet are named after legs, or hands after arms). In contrast, children with HCP performed similar to the TD 4–6 years group and thus the youngest group of control children at the beginning of their body image development. This finding might reflect differences in sensory experience between children with HCP and their TD peers, as discussed below.

### Sensory experience and lexical-semantic body representation in hemiplegic cerebral palsy children

Our data on the semantic networks for body parts in TD children suggest a developmental pattern similar to that observed previously in studies of body part identification (Witt et al., 1990; Christie and Slaughter, 2009; Camões-Costa et al., 2010; Auclair and Jambaqué, 2014). In all groups of TD children, words denoting specific body part categories (e.g., face structures, limbs, joints, internal organs) were added to the semantic network cores as age increased. Children of the group TD 4–6 years were found to primarily produce head/face structures and limbs in a non-hierarchical way (including arms, hands, legs, and feet but not dividing the upper limb into the shoulder, arm, elbow, forearm, wrist, etc.). This might reflect influences from sensorimotor afferences contributing to body schema (Christie and Slaughter, 2009). Parts of the body that receive more pronounced and early sensorimotor inputs (such as hands) may be learned preferentially (Ayres, 1961). This is substantiated by correlational analyses indicating that the body parts most frequently named by children are the structures best represented in the sensory cortex (Camões-Costa et al., 2010).

Joints were first mentioned systematically by children in the TD 7–9 years group. When reaching 7 years of age, children are in a period of consolidation and improvement of the basic patterns of movement developed as compared to early childhood (Goodway et al., 2019). In this age group, a refinement of basic motor patterns, the adaptation of motor patterns, and improvement of coordination, and motor control are observed. These new sensorimotor experiences depend on tactile, kinesthetics, proprioceptive, vestibular, and visual inputs. According to this line of reasoning, somatosensory afferences underlying body schema may also influence the development of body image at this age, improving the ability to identify and name body parts.

Only at 10–12 years did the children add internal organs and hierarchize the limbs (e.g., arm and forearm, etc.) and axial structures (e.g., neck, nape, trunk, belly, etc.). Visuospatial experience contributing to body structural description seemed to influence representations of body image at this age (Auclair and Jambaqué, 2014). Following this rationale, it seems possible that internal organs might only be learned later because they are not visible. The most salient and visible parts of the body are more identifiable and have easily observable functions and may therefore be learned before other non-visible and harder-to-experience parts of the body. Functional knowledge of some internal body parts may also emerge from formal learning about the biology of the human body (Christie and Slaughter, 2009; Auclair and Jambaqué, 2014).

The hierarchy of some axial structures (such as the division of the trunk into the neck, chest, and back) only occurs later in development. This may be due to the influence of motor learning about joints and cultural influences related to formal learning about the human body (Jaakkola and Slaughter, 2002). Studies suggest that body parts can be segmented (e.g., the arm might be considered as whole as the superior limb, or the body part joined to the forearm by the elbow) according to language, and the division of body parts can vary between different languages (Enfield, 2006; Majid, 2010). Older children are more experienced and more likely to expand their vocabulary, and the development of language is very closely related to the development of body awareness (Facon et al., 2002). Despite this, children with HCP (aged from 7 to 12 years old) presented a lexical-semantic network of body parts similar to that of the youngest TD 4–6 years group (as substantiated by Bayesian analysis). This is in line with but also expands previous studies which suggested that children with unilateral brain injury present lower performance in mental motor imagery (body schema) (Fontes et al., 2016; Di Vita et al., 2020) and also in pointing (body structural description) (Christie and Slaughter, 2009; Fontes et al., 2016) and naming (body image) (Christie and Slaughter, 2009; Fontes et al., 2016) body-part tasks than TD children.

Although joints are expected to be a part of the semantic network core of children with HCP because they were part of the semantic-lexical repertoire of children of the same age, we did not observe these children name joints in the semantic fluency task. This is an important aspect because joints are a point of reference for the segmentation of body parts, representing more detailed knowledge about the structuring of the human body (de Vignemont et al., 2009). Segmentation of the body into parts may derive from the organization of the proprioceptive and motor systems, or from perceptual factors such as the visual discontinuity of the body parts (de Vignemont et al., 2009). Following this rationale, motor activity may help to structure the mental representations of the body into functional units, according to the parts of the body that move together. In addition to representing anatomical points of reference, joints constitute the kinesiological basis of movement because the brain needs to identify the joints’ position (from a set of proprioceptive signals coming from muscles, tendons, ligaments, and joint capsule) and then plan the desired motor action (Marini et al., 2018). Difficulties in controlling movements, as often experienced by children with HCP, may thus influence their functional performance by restricting new sensorimotor experiences.

For effective motor action, for instance, when manipulating objects, it is necessary to represent the positioning and configuration of the upper limb to avoid uncomfortable or movement restrictive postures (Mutsaarts et al., 2006). Planning impairments have also been reported in young adolescents with HCP (Mutsaarts et al., 2006; Souto et al., 2020). Our study points to a delay in the development of lexical-semantic knowledge of body parts in children with HCP, which might reflect reduced sensorimotor and visuoperceptual experiences of their own bodies. Thus, it is plausible that lexical-semantic knowledge of body parts is influenced in a bottom-up manner.

When interpreting the results of the current study, some limitations have to be considered. The group of children with HCP was rather small, making it impossible to create subgroups of different ages for this group. Furthermore, it needs to be noted that the present study focused primarily on body image as only one level of body representation. In this context, it is also worth noticing that the task to evaluate participants’ body image task only drew on the semantics of body parts – which seems like a limitation in scope. Moreover, future studies might also include tasks to evaluate body schema and body structural description as well as measures for other executive function components. We used a controlled word fluency task to assess children’s knowledge of body parts. This test has a considerably higher degree of freedom compared to responses in a task requiring the naming of body parts.

Furthermore, the graph-theoretical analysis identified an emergent structure of a lexical-semantic network, qualitatively similar but less complex in children with HCP compared to TD children. Our results also suggested that the building of the lexical-semantic network for body parts and thus body image seems influenced by sensorimotor and visuoperceptual experiences. As suggested by Baumard and Osiurak (2019), bodily experience develops in everyday life under the influence of language by thinking and talking about body parts and actions. Investigations about the relationship between language and action demonstrate the involvement of motor systems in the processing of action-related language (Dalla Volta et al., 2009; Crivelli et al., 2018; Shebani and Pulvermüller, 2018). Shebani and Pulvermüller (2018) hypothesized that processing of action words semantically related to complex actions (e.g., citing “finger” and “grasping”) might facilitate elementary movements, by pre-activating a part of the movement circuit. In this context, it would also be desirable to examine if explicit conversations about body parts (“body talk”) might benefit the development of body image in children with HCP.

## Data availability statement

The original contributions presented in this study are included in the article/supplementary material, further inquiries can be directed to the corresponding author.

## Ethics statement

This study was approved by the Research Ethics Committee of the Federal University of Minas Gerais (protocol number: 2.155.379). Written informed consent to participate in this study was provided by the participants’ legal guardian/next of kin.

## Author contributions

TC, DS, PF, and VH were involved in the conceptualization and design of the original research. TC and KM were involved in the analysis. TC, VH, and KM were involved in the writing of the manuscript. All authors contributed to the revisions of the manuscript and approved the final manuscript.
